# Long-Term Usage of Breeze, a Gamified Breathing Training App, and Its Effect on Momentary Relaxation in People With Cancer: Cohort Study

**DOI:** 10.2196/70297

**Published:** 2025-12-16

**Authors:** Anja Yvonne Bischof, Tobias Budig, Sonja Schläpfer, Yanick Xavier Lukic, Fabian Schneider, Prabhakaran Santhanam, Jürgen Barth, Claudia M Witt, Tobias Kowatsch

**Affiliations:** 1 School of Medicine, University of St. Gallen, St.Gallen, Switzerland; 2 Institute for Implementation Science in Health Care, University of Zurich, Zurich, Switzerland; 3 Centre for Digital Health Interventions, Department of Management, Technology, and Economics, ETH Zurich, Weinbergstr. 56/58, Zurich, Switzerland, 41 71224 ext 7244; 4 Institute for Complementary and Integrative Medicine, University Hospital Zurich and University of Zurich, Zurich, Switzerland; 5 School of Engineering, ZHAW Zurich University of Applied Sciences, Winterthur, Switzerland; 6 Codeklang GmbH, Zurich, Switzerland; 7 Byldr GmbH, Zurich, Switzerland; 8 Datahouse AG, Zurich, Switzerland

**Keywords:** stress management, digital health, gamification, people with cancer, breathing, mindfulness, self-care, self-guided, momentary relaxation

## Abstract

**Background:**

People with cancer often experience stress. Digital health interventions can help individuals increase momentary relaxation. Breeze is a gamified breathing training that can be embedded into digital health interventions. Its effectiveness in controlled cross-sectional studies has been demonstrated. However, adherence to Breeze and its effect on momentary relaxation in longitudinal interventional studies has yet to be investigated.

**Objective:**

This study aimed to assess Breeze across 3 key dimensions: (1) its usage over time compared to other intervention components; (2) the accuracy with which users adhered to the guiding breathing pattern provided by Breeze; and (3) its effect on momentary relaxation, including the impact of the duration of the breathing session delivered by Breeze.

**Methods:**

Breeze was 1 of 7 relaxation techniques included in the CanRelax 2.0 app, designed specifically for individuals diagnosed with cancer. Participants could select any of the 7 techniques to promote momentary relaxation. The intervention was designed to last 10 weeks. However, participants were allowed to use the CanRelax 2.0 app after that period. Participants were adults diagnosed with cancer in the last 5 years recruited across Switzerland, Germany, and Austria. Adherence to the intended breathing pattern was measured using a Pearson correlation coefficient. Momentary relaxation was measured pre- and post-exercise using an 11-point visual analog scale. Statistical analyses included linear mixed effects models and calculations of effect sizes. We further analyzed the relationship between session duration and the magnitude of momentary relaxation and compared Breeze’s efficacy and time efficiency to the other available techniques.

**Results:**

Of 279 participants, 118 (42.3%) used Breeze at least once. The 118 participants engaged in 754 breathing sessions with Breeze. Momentary relaxation was assessed and calculated for 249 (33.0%) Breeze sessions. The use of Breeze declined initially but remained stable even after 10 weeks. Participants followed the predefined breathing rates of Breeze (*r*=0.9). On average, a small effect (−0.42; *P*<.001; *d*=0.19) on momentary relaxation was observed, with 2-minute breathing sessions (−0.30; *P*=.02; *d*=0.13) showing a small effect, whereas a large effect (−1.53; *P*=.03; *d*=0.74) was observed for breathing sessions of 4 minutes or longer.

**Conclusions:**

This study demonstrates the potential of Breeze to alleviate acute stress in individuals with chronic conditions, such as cancer. By combining gamification with evidence-based breathing techniques, Breeze fosters sustained user engagement and momentary relaxation. Participants adhered well to the guided breathing. While even short breathing sessions of 2 minutes provided modest momentary relaxation, longer durations (≥4 min) were considerably more effective. Future research aims to assess the impact of Breeze on other populations and chronic conditions.

## Introduction

Managing chronic conditions is complex, as it often involves high medication adherence, coping with disease progression, managing decreased quality of life, and addressing uncertainty [1-3], all of which can increase perceived stress. Therefore, incorporating regular exercises into disease management is crucial, as they help strengthen personal resilience and develop strategies to manage the chronic condition more effectively by reducing stress. Long-term elevated stress levels might emerge if mental health is not regarded in people with a chronic condition. Chronic stress characterizes itself as a prolonged period of challenging or threatening life conditions that interfere with daily life and last for a longer period, that is, for a minimum of 1 month [4]. People dealing with chronic stress face a higher risk of developing further chronic conditions, a higher risk of mortality, and faster biological aging [5-7].

Approaches for stress reduction in people with chronic conditions, such as cancer, are manifold. Some of these are meditation (eg, short meditation, mindfulness meditation, walking meditation, or body scan), formal relaxation techniques (eg, guided imagery and progressive muscle relaxation), or breathing exercises [1,8-12]. Different types of techniques and exercises may have a positive impact on reduced worry and psychological stress [13,14]. To reduce acute periods of stress, in particular, slow-paced breathing training shows positive effects on physiological markers and psychological well-being [15]. It further reduces chronic pain [16] and improves dealing with acute and chronic stress [15,17]. Reducing the impact of daily acute stressors may have a positive effect on the reduction of chronic stress as well [18].

Digital health interventions (DHIs) can support individuals with chronic conditions in consistently engaging in stress-reducing exercises, helping to lower stress levels and enhance resilience [19,20]. One approach is *Breeze* (research prototype co-developed by YXL, Helen Galliker, and TK), a smartphone-based gamified breathing training that uses breathing sounds as biofeedback to guide users through a slow-paced breathing training. In experimental cross-sectional studies and online experiments, Breeze has shown its potential for stress reduction [21-23]. Slow-paced, controlled breathing has the potential to achieve momentary relaxation [12].

As promising as DHIs are, one major challenge is low adherence [24-26]. Studies show dropout rates of 50% to 83% in DHIs for various chronic conditions [25,27-29]. Adherence is a prerequisite to achieving intended health outcomes [30]. Gamification can enhance the user experience with a DHI and, thus, may address low adherence rates [31,32].

However, long-term use and effectiveness in a longitudinal study assessing a gamified breathing training with people with chronic conditions are still open. To this end, we formulate the following 3 research questions (RQs): (RQ1) How often is Breeze used over time? (RQ2) How accurately do participants adhere to the guided breathing rate of Breeze? and (RQ3) What is the effect of Breeze on momentary relaxation?

## Methods

### Overview

To answer the research questions, this study leverages usage data of the smartphone-based CanRelax 2.0 app (research prototype developed by the study team) [33,34], which was recently evaluated in a randomized controlled trial (RCT; German Clinical Trials Register DRKS00027546; registered on February 23, 2022). The RCT with a third randomized arm examined the effectiveness of CanRelax 2.0 in reducing stress among people with cancer over 10 weeks. The participants were allowed to use the CanRelax 2.0 app even after the end of the study intervention period. The app contained 7 different relaxation techniques, including Breeze. Participants were able to self-select exercises, and no exercise was highlighted. Where applicable, the study adhered to STROBE (Strengthening the Reporting of Observational Studies in Epidemiology) guidelines. Detailed information on the RCT procedures and the CanRelax 2.0 app has been published [33-35].

### Recruitment

Participants were recruited between July 2022 and February 2023 in Switzerland, Germany, and Austria. Recruitment efforts included social media posts (via Facebook, LinkedIn, and X [formerly Twitter]) and traditional outreach methods, such as consultations with health care providers, printed flyers, newsletters, and a press release issued by the University Hospital Zurich. A dedicated project website provided essential study information and app store download links to support recruitment. Eligibility criteria included being at least 18 years old, fluent in German, owning a smartphone (Android or iOS), and having received a cancer diagnosis within the past 5 years. Exclusion criteria included self-reported suicidal ideation and pregnancy at baseline. Further information on the recruitment strategy was published previously [33,34].

### Intervention

**Figure 1.**
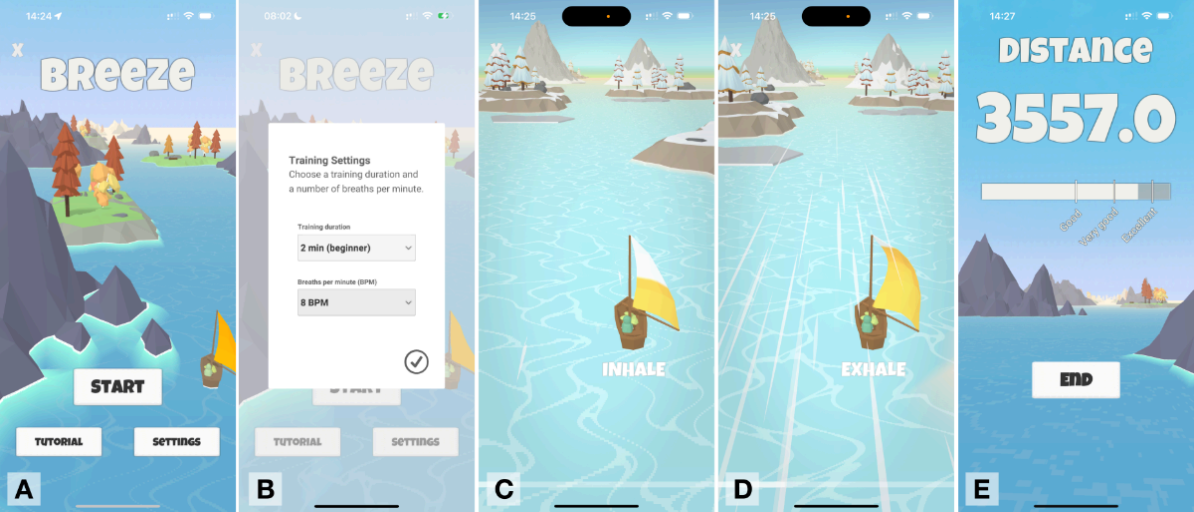
One entire session of Breeze. (A) The home screen offers 3 primary options: initiating the Breeze session directly, accessing a tutorial on how to use the Breeze application, or adjusting the breathing session settings; (**B**) In the settings section, participants can set the training duration and the breaths per minute; (**C, D**) Textual and visual feedback is provided to guide participants through the breathing training; (**E**) On completing the session, the distance covered by the sailing boat is displayed as a performance metric, serving as an indicator of the participant’s adherence to the guided breathing instructions. The study was conducted using a German-language interface.

The breathing training Breeze was 1 of 7 distinct relaxation exercises integrated into the CanRelax 2.0 app [33,34]. Breeze supports slow-paced breathing in a gamified setting, where the smartphone’s microphone is used to continuously detect breathing phases in quasi-real time to accelerate a virtual sailboat if an individual is exhaling through the mouth [21,36]. A visual animation in the sail provides guidance according to a slow-paced breathing pattern (8 breaths per minute, the default setting; the breathing rhythm consists of inhale, pause, exhale). Breath detection has been proven to be highly accurate [23]. Figure 1 provides an overview of a slow-paced breathing session of Breeze. The intended usage pattern was 3 relaxation exercises per week, which participants could adjust by self-set goals, meaning that some weeks may have been completed without using Breeze.

Consistent with prior work [34], momentary relaxation was assessed using an 11-point visual analog scale (0=very relaxed and 10=very tense) before and after every third time a participant used any of the relaxation exercises included in the CanRelax 2.0 app. Momentary relaxation change was then calculated as the difference between the pre- and post-exercise values of perceived momentary relaxation. The default duration of the breathing exercise was set to 2 minutes, and the sailboat animation guided individuals to follow 8 breaths per minute. Although slow-paced breathing training is characterized as being especially effective with 6 breaths per minute [17], many people are not able to apply this rate and need to train toward it. Participants were able to change the duration and the guided breathing rate according to personal preferences. Breeze incorporates a concise, slide-based tutorial designed to familiarize users with the benefits of slow-paced breathing training while providing a brief overview of its guidance and interaction features. On initial access, the start button on the home screen was inactive. Completion of the tutorial activated the start button, allowing users to initiate their first training session [23].

### Data Preparation

The CanRelax 2.0 app collected demographic information, momentary relaxation levels, app usage metrics, and additional information required for the RCT. For this work, we focused exclusively on data related to the intervention component Breeze. We extracted the timestamp of the exercise, its duration, and perceived momentary relaxation before and after the exercises. Moreover, we used the breathing detection classifier of Breeze 2 to measure the accuracy of the breathing rate [23].

The initial dataset comprised 787 Breeze sessions and 265 pairs of pre- and post-exercise values of participants’ momentary relaxation, as only every third session was evaluated to minimize survey fatigue in the whole CanRelax study. We applied a series of exclusion criteria to ensure data quality and reliability. First, 2 pre- and post-exercise values of participants’ momentary relaxation were removed, as they lacked either a pre- or post-exercise value (eg, Breeze session not completed). Additionally, 11 pairs of pre- and post-exercise values were excluded due to incomplete datasets, which made it impossible to retrieve essential exercise details (eg, duration). Moreover, 7 exercise sessions were excluded where user IDs were missing, as these were crucial for linking data across sessions. Furthermore, a duration-based outlier criterion was applied to remove exercise sessions that were either shorter than 30 seconds or longer than 1000 seconds, resulting in the exclusion of 13 exercise sessions. After these exclusions, the dataset consisted of 754 (754/787, 95.8%) Breeze sessions and 249 (249/265, 93.9%) pairs of pre- and post-exercise values.

### Statistical Analysis

#### Descriptive Statistics

During participant registration, demographic data on sex, age, and education were collected. The use of CanRelax 2.0 relaxation exercises was tracked, enabling the calculation of each user’s activity. In general, a participant is a person who was part of the study; an active user refers to an individual who performed at least one Breeze session within the corresponding time frame (eg, the entire study, a specific week).

#### Usage of Breeze Over Time

To assess potential differences between participants who engaged with the Breeze application (Breeze users) and those who did not (nonusers), a series of post hoc comparative analyses were conducted. Demographic characteristics, including sex, education level (nontertiary, tertiary), and age group (18‐44, 45‐64, and ≥65 years), were compared between Breeze users and nonusers using chi-squared tests. Furthermore, overall app engagement, quantified by the average weekly number of all exercises performed, was compared between Breeze users and nonusers using a Welch *t* test. A *P* value of <.05 was considered statistically significant for all analyses.

For Breeze usage over time, we evaluated the recorded sessions over time. Each participant had a timestamp of the first session, which was used to calculate the relative day of each session. Moreover, the self-adjusted target for exercises was used to calculate the weekly share of Breeze. To compare the user engagement distribution before and after the intervention period (weeks >10), 2 metrics were used. First, the weekly number of distinct participants was tracked over time. Second, patient retention was assessed by comparing the number of participants from a given week (week 1) who reappeared in a subsequent week (week 2). This comparison generated a matrix illustrating the return rate of users from a source week (week 1) in a future week (week 2).

#### Breathing Adherence

Participant adherence to the guided breathing rate was assessed for each Breeze session. The application’s validated sound detection algorithm [23] provided an estimated performed breathing rate. Adherence was then analyzed by (1) visually comparing distributions of measured breathing rates against the intended (user-selected) breathing rates via box plots, categorized by the intended rate; and (2) quantitatively assessing the relationship through a linear regression fit of measured rates on intended rates, supplemented by the Pearson correlation coefficient between the two.

#### Effect on Momentary Relaxation

To quantify the impact of the breathing exercise on momentary relaxation, a linear mixed effects fit was performed. This approach was chosen because the design involved repeated measures, with each participant providing momentary relaxation ratings at 2 time points (pre- and post-exercise). Mixed effects modeling allows us to account for within-session variability by including random intercepts for each session, thereby controlling for baseline differences in stress levels [37].

Each measured exercise in the cleaned dataset includes a pair of pre- and post-exercise values for the participant’s momentary relaxation. To estimate the average change in momentary relaxation following the intervention, we fit a linear mixed effects model. The model included a dummy-coded fixed effect for the measurement time point (is_post: 0=preexercise and 1=postexercise). To account for repeated measures within the same exercise session, we included a random intercept for *experiment_id*. This resulted in the Model 1: *Perceived_stress_measure ~ is_post+(1 | experiment_id*).

For the implementation [38], the Python (version 3.12.7) package statsmodels (version 0.14.2) [39] was used. The linear fixed effect model calculations in the package are based on Lindstrom et al [40].

Moreover, a second model was used to investigate the effect of other covariates. The outcome (*momentary relaxation change*) was defined as the difference between the pre- and post-exercise values of participants’ perceived momentary relaxation. Lower values on this scale indicate a greater increase in relaxation, reflecting the intended effect of the intervention. It included the number of Breeze exercises already practiced by the person (*usage_count*), the duration of Breeze (*duration_minutes*), the average use of Breeze per week (*avg_weekly_earned*), the measured breathing rate of Breeze (*breathing_rate),* and the number of days the participant had participated in the study up to that point (*relative_day*). The usage count, average weekly use, and number of relative days in the study were considered potential indicators of a learning effect during the observed period. Additionally, duration and breathing rate of the exercise were hypothesized to influence the momentary relaxation change directly. To this end, model 2 is formulated as follows: *momentary relaxation change ~ usage_count+duration_minutes + avg_weekly_earned+ breathing_rate+relative_day+(1 | experiment_id*).

The results were evaluated based on the *P* value of each variable during a test for significance. Here, a 5% threshold was applied [41]. To assess the effect size of Breeze to promote momentary relaxation (see RQ3), we calculated Cohen *d* by the method proposed by Westfall et al [42] as Brysbaert and Stevens [43] recommended for linear mixed effect models. To this end, it was computed by dividing the fixed effect coefficient by the square root of the sum of the random intercept variance and residual variance, accounting for both between-subject and within-subject variability in the mixed effects framework.

### Ethical Considerations

The trial was conducted in accordance with the principles of the Declaration of Helsinki. The Ethics Committee of Zurich, Switzerland, reviewed the study synopsis and concluded that the project does not fall under the Swiss Human Research Act (BASEC 2021-01071); therefore, a full ethics review was not required. All participants were adults (aged ≥18 y) and provided informed electronic consent within the app. Participants were informed about the study purpose, the voluntary nature of participation, the data collected through the app, and their right to withdraw at any time without negative consequences. Neither the study team nor the CanRelax 2.0 app requested any personally identifiable information at any stage of the study. Participants could provide a (fictional) nickname to be used by the conversational agent of the CanRelax 2.0 app. App-derived and survey data were stored on secure, access-restricted servers. All data handling complied with applicable Swiss data protection regulations. Participants did not receive any financial compensation for their participation, but the app was free of charge. The study was registered in the German Clinical Trials Register (DRKS00027546) on February 23, 2022. The first participant was enrolled on July 20, 2022, and the last app session took place on September 30, 2023.

## Results

### Descriptive Statistics

Assessments of momentary relaxation were collected both pre- and post-exercise for 249 (33.0%) of the sessions. Table 1 summarizes the demographic characteristics of the participants, whereas Table 2 provides an overview of engagement with the intervention components.

Analyses comparing Breeze users and nonusers revealed no statistically significant differences in terms of sex (*χ*^2^_1_=0.04; *P*=.85), age group (*χ*^2^_2_=0.2; *P*=.90), education level (*χ*^2^_1_=2.8; *P*=.10), and distress before study start (*t*_1_=0.66; *P*=.51). Moreover, Breeze users performed a significantly higher average weekly number of all exercises (mean 3.85, SD 3.32) compared to nonusers (mean 1.99, SD 2.04; *t*_1_=5.07; *P*<.001), indicating that Breeze use co-occurred with higher general app activity.

**Table 1.** Descriptive statistics for the 118 participants who used Breeze at least once.

Characteristics	Total number, n (%)
Sex assigned at birth (female)	96 (81.4)
Age (y)	
18‐44	15 (12.7)
45‐64	84 (71.2)
>64	19 (16.1)
Educational attainment	
Nontertiary	28 (23.7)
Tertiary	56 (47.5)
Missing	34 (28.8)
Number of completed Breeze sessions	754 (100)
Number of Breeze sessions with pre- and post-exercise relaxation values	249 (33.0)

**Table 2.** Descriptive statistics of Breeze usage.

Characteristic	Values, mean (SD)	Minimum	Maximum
Total number of Breeze sessions per Breeze users	6.39 (10.61)	1	92
Duration of a Breeze session (sec)	150 (41)	31	376
Preexercise value of momentary relaxation	4.59 (2.15)	1	10
Postexercise value of momentary relaxation	4.17 (2.35)	0	10
Momentary relaxation after having used Breeze^a^	−0.42 (1.67)	–6	7

a Calculated as the difference between the post- and preexercise value of momentary relaxation.

### How Often Is Breeze Used Over Time?

**Figure 2.**
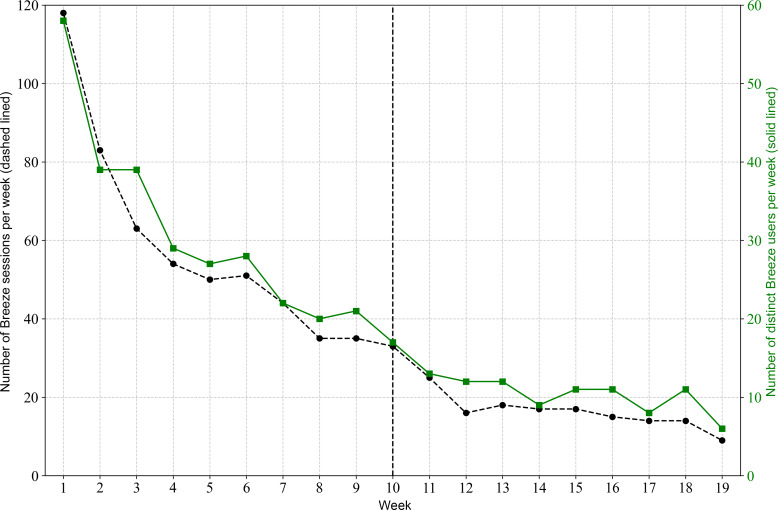
Number of Breeze sessions per week over time (dashed line) and number of distinct users per week who used Breeze in that week (solid line). The dotted line at week 10 indicates the end of the study intervention period; participants were allowed to use the CanRelax 2.0 app afterward.

**Figure 3.**
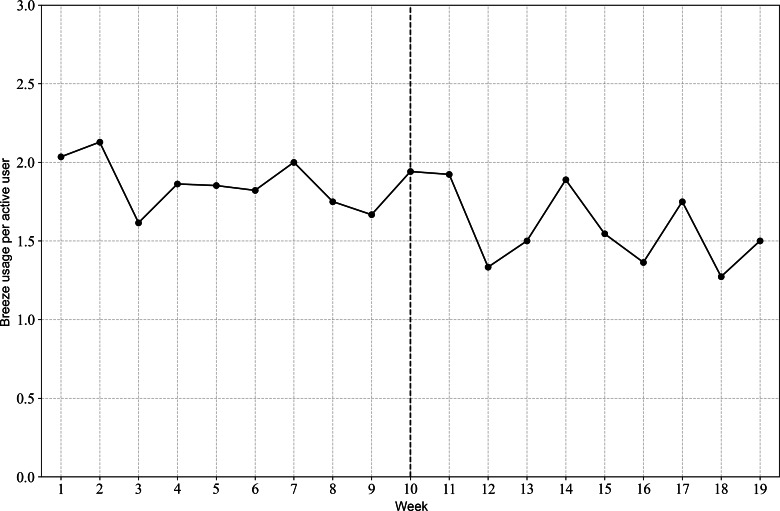
Average weekly Breeze sessions by active Breeze users.

**Figure 4.**
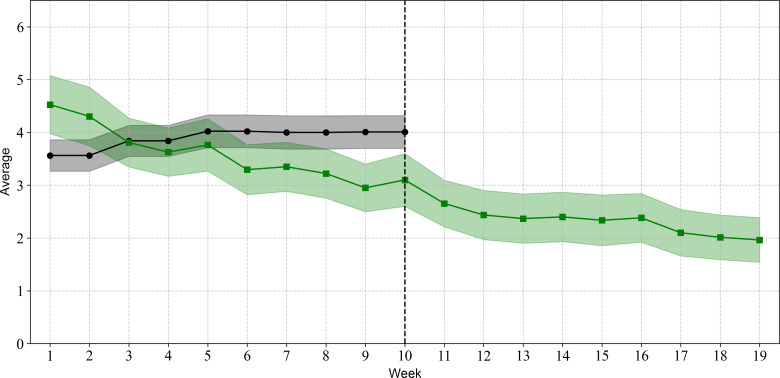
Average weekly relaxation goal (black) and average number of relaxation exercises practiced per participant per week (green; all types of relaxation techniques). Note: The shadow represents the SDs. The dotted line at week 10 indicates the end of the study intervention period; participants were allowed to use the CanRelax 2.0 app afterward. However, from week 10 onward, the weekly relaxation goals could no longer be adjusted.

Breeze usage is shown in Figures2^5^. Figures2  3 illustrate the relationship between the number of Breeze sessions per week and the number of distinct Breeze users per week. While Figure 2 shows a decline in absolute numbers over time, Figure 3 demonstrates that the number of Breeze sessions per user remained relatively stable, fluctuating between 1.5 and 2 sessions per user throughout the study period. Figures4  5 compare the usage of Breeze with the other 6 relaxation techniques available in the CanRelax 2.0 app. During weeks 1 to 3, participants exceeded the target number of exercises; however, this trend did not persist for the remainder of the intervention period. After the intervention ended, participants continued to use the CanRelax 2.0 app, but the average number of only Breeze exercises performed declined to approximately 2 per week. Notably, the proportion of Breeze sessions relative to all relaxation techniques in the app decreased over time.

**Figure 5.**
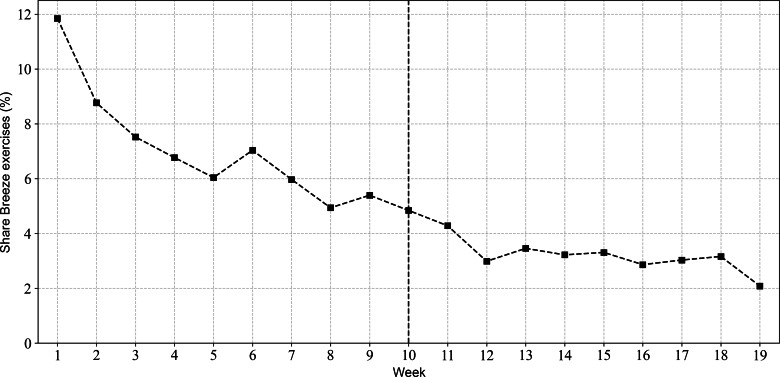
Share of practiced Breeze exercises relative to all 7 relaxation techniques.

For Breeze usage after the intervention period (weeks >10), user engagement stabilized at approximately 10.3 (SD 1.5) distinct users per week (Figure 2). A cohort analysis (Multimedia Appendix 1) further revealed that approximately 4.2 (SD 1.1) of these were returning users. This suggests a user base comprising a stable core alongside a broader, more sporadic group without a dominant trend toward either.

### How Accurately Do Participants Adhere to the Guided Breathing Rate of Breeze?

**Figure 6.**
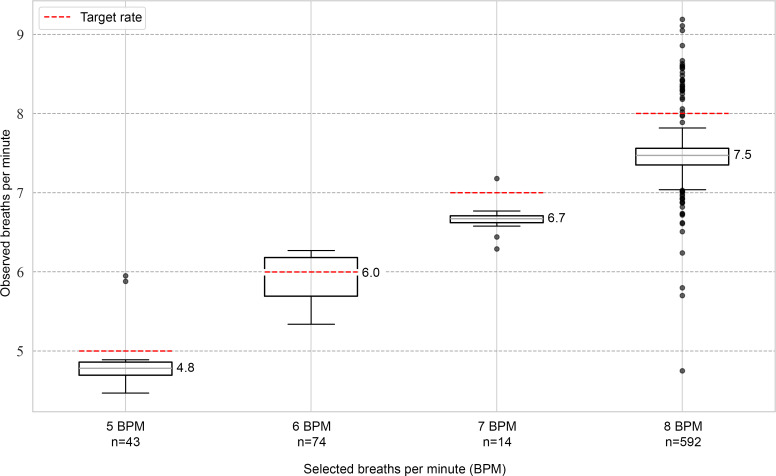
Boxplot of measured breathing rate according to the selected breathing rate. Horizontal dashed lines indicate the selected (target) breathing rate for each group. The number next to the box plot shows the median of that group. Note: only 723 (95.9%) of 754 sessions are shown; the remaining 4.1% either had more or fewer breaths per minute selected and are thus not displayed.

Figure 6 shows the relationship between the measured breathing rate and the visually guided breathing rate selected by the Breeze users. In most sessions (592/754, 78.5%), users chose the default breathing rate of 8 breaths per minute.

A linear fit of the measured versus selected breathing rates indicates an *R*^2^ of 0.81 and a root mean squared error of 0.38 breaths per minute. Moreover, the Pearson coefficient for the correlation is 0.9. These results indicate that Breeze guided participants well with visual cues and acoustic biofeedback.

### What Is the Effect of Breeze on Momentary Relaxation?

**Figure 7.**
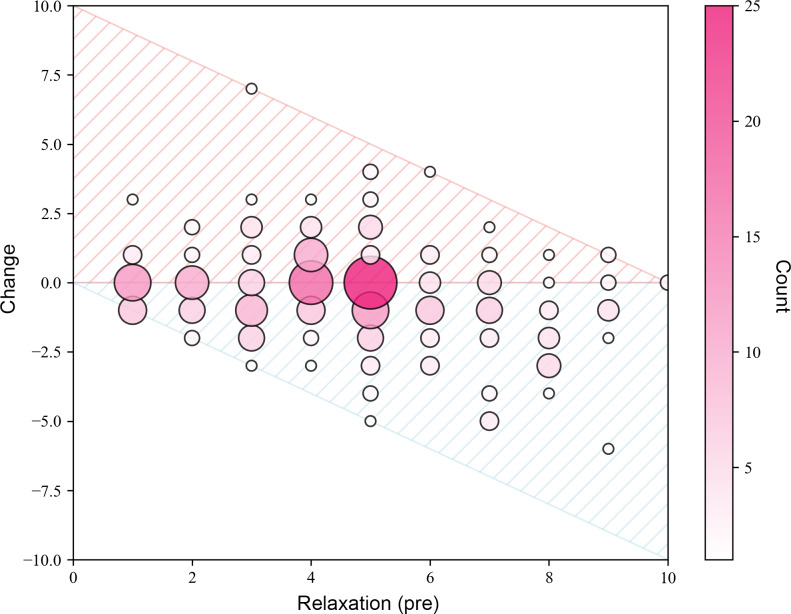
Scatter plot illustrating the distribution of momentary relaxation changes from before to after the breathing exercise. Note: The x-axis represents participants’ momentary relaxation before using Breeze. The y-axis shows the change in momentary relaxation between post- and pre-exercise ratings. A negative change indicates an increase in participants’ relaxation after using Breeze.

**Figure 8.**
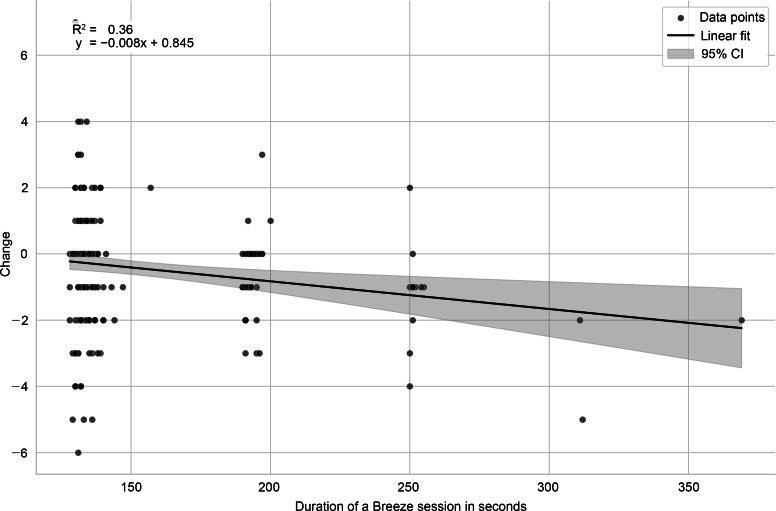
Scatter plot of relaxation change (post-pre) against breathing duration together with linear fit. Note: The x-axis represents the duration of the Breeze session. The y-axis represents the change between the post- and pre-exercise value of participants’ momentary relaxation. A negative change indicates an increase in participants’ relaxation after using Breeze.

Figure 7 illustrates the distribution of momentary relaxation changes for all measurements given the preexercise value. One finding is that higher preexercise distress values are linked to a greater change in momentary relaxation (Pearson correlation −0.26). In a first linear fixed-effects model with the experiment treated as a random effect, performing a Breeze exercise was associated with a significant decrease in the outcome (estimate −0.42; SE 0.11; *P*<.001), corresponding to a small effect size (Cohen *d*=0.19). In the covariate analysis (model 2), the duration of Breeze sessions was the only significant predictor of the outcome (Table 3). Longer Breeze sessions were associated with a reduction in the outcome (−0.56; SE 0.17; *P*=.002), corresponding to a small-to-medium effect (Cohen *d*=0.31). None of the other covariates, including the number of previously practiced Breeze exercises, average Breeze use per week, measured breathing rate, or number of days participated in the study, were significantly associated with the outcome (*P* values >.05). Plotting the momentary relaxation change against the exercise duration (Figure 8) suggests a favorable effect of longer durations as illustrated by Table 4, which lists effect sizes for 2, 3, and ≥4 minutes of Breeze’s exercise durations. The results indicate that longer exercise durations increase the changes.

**Table 3.** Results of the investigation of covariates with model 2.

Variable	Estimate	SE	*P* value	Cohen *d^a^*
Number of Breeze exercises already practiced by the person	−0.007	0.008	.41	N/A^b^
Duration of Breeze	−0.557	0.172	.002	0.31
Average use of Breeze per week	−0.029	0.026	.27	N/A
Measured breathing rate guided by Breeze	0.023	0.135	.87	N/A
Number of days participated in the study	−0.001	0.002	.52	N/A

a Cohen *d* is only reported if the level of significance is ≤.05.

b N/A: not applicable.

**Table 4.** Summary of stress reduction effects by breathing exercise duration.

Duration	Effect estimate	SE	*P* value	Cohen *d*	Observations	Unique users
2 min	−0.30	0.13	.017	0.13	382	68
3 min	−0.56	0.18	.035	0.26	86	19
4+ min	−1.53	0.42	.028	0.74	30	8

## Discussion

### Principal Findings

In this study, 3 dimensions of Breeze as a tool for helping participants with chronic conditions manage stress were assessed: its usage over time, the accuracy with which participants adhered to the visually guided, slow-paced breathing rate, and its effect on momentary relaxation. The analysis of Breeze usage over time demonstrated that the number of Breeze evaluations was comparable to that of the other 6 relaxation techniques included in the CanRelax 2.0 app. Although the intervention ended after 10 weeks, the participants were allowed to use the CanRelax 2.0 beyond the end of the study intervention period, and some users continued engaging with Breeze. This sustained usage suggests that the intervention effectively promotes long-term user engagement.

Accuracy in adhering to the preset breathing rate was also a key focus, as precise use is critical for achieving optimal relaxation. The default breathing rate was set to 8 breaths per minute. Participants could adjust this rate based on personal preference. As shown in Figure 6, the 8-breaths-per-minute rate was selected in most Breeze sessions (592/754, 78.5%). Regardless of the breathing rate, the Pearson correlation coefficient for participants’ accuracy in following the breathing rate guided by Breeze is 0.9. This high adherence to the target rate suggests that the exercise is intuitively designed and easy to follow.

Regarding momentary relaxation, findings indicated larger effects in participants with higher preexercise values of perceived stress. Although relaxation effects were also observed in participants with lower stress levels, the effect size was smaller. This pattern suggests that Breeze effectively reduces momentary stress in people with cancer, underscoring the intervention’s relevance in high-stress scenarios.

Finally, the relationship between Breeze session duration and its impact on momentary relaxation was examined. The results indicated that relaxation effects were associated with session duration, with participants who chose longer sessions reporting larger stress reduction. While the default 2-minute duration was associated with a stress reduction effect of −0.30, sessions of 4 minutes or more were associated with an effect of −1.53. These findings support Breeze as a low-burden intervention capable of delivering immediate momentary relaxation.

When comparing the results of Breeze with the 6 other relaxation techniques included in the CanRelax 2.0 app [34], the momentary relaxation effect of a 2- or 3-minute Breeze exercise initially appears modest (a change of −0.4). It ranked seventh in absolute effect, with walking meditation (−0.6) being the second least effective and progressive muscle relaxation the most effective (−1.45). However, this comparison must be contextualized by the significantly different time investments required. Breeze sessions are exceptionally brief, with an average duration of 2.5 minutes, whereas other techniques require substantially more time, ranging from 5 to nearly 40 minutes (Multimedia Appendix 2).

When the relaxation effect was analyzed per minute of engagement, Breeze demonstrated a high level of efficiency. It was associated with a relaxation effect of 0.16 units per minute, a rate comparable to longer exercises, such as walking meditation (0.12 units per min) and progressive muscle relaxation (0.12 units per min).

Furthermore, if users chose Breeze sessions of 4 minutes or longer, the associated effect of −1.53 was the highest observed among all techniques in the study. This finding is particularly important because a 4-minute session remains shorter than any other relaxation technique offered by the CanRelax 2 app. Collectively, these results indicate that Breeze is not only a highly time-efficient intervention but also has the potential to deliver superior relaxation effects with only a modest increase in duration.

### Limitations

This study presents 4 main limitations. First, Breeze was embedded within the CanRelax 2.0 app, offering 6 additional relaxation techniques [33,34]. This design allowed participants to select techniques based on their individual preferences, meaning some participants may never have used Breeze, resulting in a lack of data on these individuals. However, participants who chose to use Breeze did so voluntarily, without being specifically encouraged to select it. Despite this, the study demonstrated consistent engagement with Breeze among its users, maintaining a stable user base throughout the 10-week intervention period and beyond.

Second, momentary relaxation was assessed using a single-item scale, whereas an internationally validated, standardized evaluation scale, such as the Relaxation State Questionnaire [44], could have provided more detailed insights into the relaxation effects. However, given the study’s goal to minimize participant burden, the single-item scale was appropriate and sufficient for capturing the momentary relaxation effects.

Third, the observed pattern of larger effects in participants with higher preexercise stress levels may be partially explained by regression to the mean. Participants are likely to use Breeze when experiencing elevated stress, and subsequent measurements naturally tend to return closer to their average baseline state, independent of the intervention’s actual effect. While the data are consistent with Breeze providing stress reduction, this statistical phenomenon represents an alternative explanation that should be considered when interpreting the relationship between preexercise stress levels and relaxation outcomes.

Finally, findings indicated that longer Breeze sessions were associated with larger momentary relaxation effects, with sessions lasting 4 minutes or longer showing a significant decrease in stress. Hypothetically, participants who extended their session duration may have been more motivated, potentially leading to larger effects. However, this study did not assess whether elevated motivation directly contributes to increased momentary relaxation, nor did it examine the isolated effect of exercise duration on momentary relaxation. Future studies should further investigate the relationship between session duration and the momentary relaxation effects to confirm whether an extended session time consistently enhances relaxation outcomes across a broader sample.

### Comparison With Prior Work

To the best of our knowledge, this is the first study that applies a gamified, biofeedback-guided breathing exercise in a real-world setting, where the exercise is available in a study conducted over a 10-week period [33,34]. Until now, Breeze has only been tested in a controlled setting, where its effectiveness has been shown [23].

The usage of Breeze over time was comparable to the other 6 relaxation techniques in the CanRelax 2.0 app. Additionally, the high Pearson correlation for participants’ accuracy in following the breathing rate guided by Breeze highlights its intuitive use and the easy-to-understand approach – both key factors for adopting a DHI [45,46]. Furthermore, the gamified approach may help sustain participants’ motivation over several weeks. Gamification offers a unique way for users to practice and refine skills in a safe, interactive environment, as seen in Breeze. It provides both extrinsic motivation (eg, by measuring the distance traveled with the sailing boat) and fosters intrinsic motivation through visible progress (eg, by seeing the wind fill the boat’s sail when exhaling at the right time) [47]. By incorporating user control elements (ie, setting an individual duration and breathing rate), gamified interventions can enhance a sense of autonomy [48], while facilitating flow or immersion—key factors for enjoyment and sustained attention [47] to achieve higher and longer-lasting use.

Momentary relaxation is especially important for people dealing with chronic conditions, such as cancer. Cancer and its treatment may lead to increased levels of anxiety, fatigue, and physical discomfort, which can worsen overall quality of life [2,3]. Momentary relaxation provides temporary but effective relief from these stressors, helping to improve emotional well-being [49,50]. Moments of relaxation can also support building resilience and promote a more adaptive response to the challenges of chronic conditions [50]. Thus, Breeze might be an effective intervention component for various DHIs aiming to reduce acute stress.

Breathing exercises are an effective intervention for reducing stress [12]. Deep, controlled breathing slows the heart rate, lowers blood pressure, and reduces levels of stress hormones like cortisol [12,51,52]–leading to a perceived reduction in acute stress. Additionally, breathing exercises calm the mind, which can positively impact interrupting cycles of anxiety or negative thinking, leading to a calmer mental state and improved emotional regulation [51-53]. Practicing regular breathing techniques with Breeze might, therefore, have the potential to increase resilience to stress and improve overall well-being. Breeze may therefore also offer benefits to individuals coping with other conditions, such as depression, anxiety, hypertension, or type 2 diabetes.

### Conclusions

The study demonstrates that Breeze has the potential to effectively increase momentary relaxation among people with cancer. This finding supports the suitability of DHIs as a tool to assist individuals with chronic illnesses, providing them with various relaxation techniques to help manage daily challenges. Additionally, participants used the option of extending session duration and adjusting the breathing rate according to personal preferences. This suggests that future relaxation interventions may benefit from allowing users to customize settings, thereby meeting diverse needs within a single application.

## Supplementary material

10.2196/70297 Multimedia Appendix 1 Cohort analysis.

10.2196/70297 Multimedia Appendix 2 Momentary relaxation effect of non-Breeze exercises.

## Abbreviations

DHI: digital health intervention
RCT: randomized controlled trial
RQ: research question
STROBE: Strengthening the Reporting of Observational Studies in Epidemiology

## References

[1] Weis J (2015). Psychosocial care for cancer patients. Breast Care (Basel).

[2] Antoni MH, Dhabhar FS (2019). The impact of psychosocial stress and stress management on immune responses in patients with cancer. Cancer.

[3] Gil F, Costa G, Hilker I, Benito L (2012). First anxiety, afterwards depression: psychological distress in cancer patients at diagnosis and after medical treatment. Stress Health.

[4] Crosswell AD, Lockwood KG (2020). Best practices for stress measurement: how to measure psychological stress in health research. Health Psychol Open.

[5] Nyberg ST, Fransson EI, Heikkilä K (2013). Job strain and cardiovascular disease risk factors: meta-analysis of individual-participant data from 47,000 men and women. PLoS ONE.

[6] Holt-Lunstad J, Smith TB, Baker M, Harris T, Stephenson D (2015). Loneliness and social isolation as risk factors for mortality: a meta-analytic review. Perspect Psychol Sci.

[7] Epel ES, Crosswell AD, Mayer SE (2018). More than a feeling: a unified view of stress measurement for population science. Front Neuroendocrinol.

[8] Carlson LE (2023). Psychosocial and integrative oncology: interventions across the disease trajectory. Annu Rev Psychol.

[9] Psychoonkologische diagnostik, beratung und behandlung von erwachsenen krebspatient innen. Leitlinienprogramm Onkologie.

[10] Riba MB, Donovan KA, Andersen B (2019). Distress Management, Version 3.2019, NCCN Clinical Practice Guidelines in Oncology. J Natl Compr Canc Netw.

[11] Dehghan M, Jazinizade M, Malakoutikhah A (2020). Stress and quality of life of patients with cancer: the mediating role of mindfulness. J Oncol.

[12] Balban MY, Neri E, Kogon MM (2023). Brief structured respiration practices enhance mood and reduce physiological arousal. Cell Rep Med.

[13] Santos F dos, Lacerda SS, Coelhoso CC, Barrichello CR, Tobo PR, Kozasa EH (2021). The integration of meditation and positive psychology practices to relieve stress in women workers (flourish): effects in two pilot studies. Behav Sci (Basel).

[14] Gao L, Curtiss J, Liu X, Hofmann SG (2018). Differential treatment mechanisms in mindfulness meditation and progressive muscle relaxation. Mindfulness (N Y).

[15] Goessl VC, Curtiss JE, Hofmann SG (2017). The effect of heart rate variability biofeedback training on stress and anxiety: a meta-analysis. Psychol Med.

[16] Chittaro L, Sioni R (2014). Evaluating mobile apps for breathing training: the effectiveness of visualization. Comput Human Behav.

[17] Russell MEB, Scott AB, Boggero IA, Carlson CR (2017). Inclusion of a rest period in diaphragmatic breathing increases high frequency heart rate variability: implications for behavioral therapy. Psychophysiology.

[18] O’Connor DB, Thayer JF, Vedhara K (2021). Stress and health: a review of psychobiological processes. Annu Rev Psychol.

[19] Ang WHD, Chew HSJ, Dong J, Yi H, Mahendren R, Lau Y (2022). Digital training for building resilience: systematic review, meta-analysis, and meta-regression. Stress Health.

[20] Schäfer SK, von Boros L, Schaubruch LM (2024). Digital interventions to promote psychological resilience: a systematic review and meta-analysis. NPJ Digit Med.

[21] Shih CH, Tomita N, Lukic YX, Reguera ÁH, Fleisch E, Breeze KT (2019). Smartphone-based acoustic real-time detection of breathing phases for a gamified biofeedback breathing training. Proc ACM Interact Mob Wearable Ubiquitous Technol.

[22] Lukic YX, Klein SS, Brügger V, Keller OC, Fleisch E, Kowatsch T (2021). The impact of a gameful breathing training visualization on intrinsic experiential value, perceived effectiveness, and engagement intentions: between-subject online experiment. JMIR Serious Games.

[23] Lukic YX, Teepe GW, Fleisch E, Kowatsch T (2022). Breathing as an input modality in a gameful breathing training app. JMIR Serious Games.

[24] Meyerowitz-Katz G, Ravi S, Arnolda L, Feng X, Maberly G, Astell-Burt T (2020). Rates of attrition and dropout in app-based interventions for chronic disease: systematic review and meta-analysis. J Med Internet Res.

[25] Torous J, Lipschitz J, Ng M, Firth J (2020). Dropout rates in clinical trials of smartphone apps for depressive symptoms: A systematic review and meta-analysis. J Affect Disord.

[26] Pratap A, Neto EC, Snyder P (2020). Indicators of retention in remote digital health studies: a cross-study evaluation of 100,000 participants. NPJ Digit Med.

[27] Lie SS, Karlsen B, Oord ER, Graue M, Oftedal B (2017). Dropout from an eHealth intervention for adults with type 2 diabetes: a qualitative study. J Med Internet Res.

[28] Melville KM, Casey LM, Kavanagh DJ (2010). Dropout from internet-based treatment for psychological disorders. Br J Clin Psychol.

[29] Christensen H, Griffiths KM, Farrer L (2009). Adherence in internet interventions for anxiety and depression. J Med Internet Res.

[30] Yardley L, Spring BJ, Riper H (2016). Understanding and promoting effective engagement with digital behavior change interventions. Am J Prev Med.

[31] Jakob R, Harperink S, Rudolf AM (2022). Factors influencing adherence to mHealth apps for prevention or management of noncommunicable diseases: systematic review. J Med Internet Res.

[32] Castro O, Mair JL, Salamanca-Sanabria A (2023). Development of “LvL UP 1.0”: a smartphone-based, conversational agent-delivered holistic lifestyle intervention for the prevention of non-communicable diseases and common mental disorders. Front Digit Health.

[33] Schläpfer S, Schneider F, Santhanam P (2024). Engagement with a relaxation and mindfulness mobile app among people with cancer: exploratory analysis of use data and self-reports from a randomized controlled trial. JMIR Cancer.

[34] Schläpfer S, Astakhov G, Pawel S (2024). Effects of app-based relaxation techniques on perceived momentary relaxation: observational data analysis in people with cancer. J Psychosom Res.

[35] Barth J, Schläpfer S, Schneider F (2025). Mobile health intervention CanRelax reduces distress in people with cancer in a randomized controlled trial. NPJ Digit Med.

[36] Lin IM, Tai LY, Fan SY (2014). Breathing at a rate of 5.5 breaths per minute with equal inhalation-to-exhalation ratio increases heart rate variability. Int J Psychophysiol.

[37] Hilbert S, Stadler M, Lindl A, Naumann F, Bühner M (2019). Analyzing longitudinal intervention studies with linear mixed models. TPM Test Psychom Methodol Appl Psychol.

[38] Bates D, Mächler M, Bolker B, Walker S (2015). Fitting linear mixed-effects models using lme4. J Stat Softw.

[39] Seabold S, Perktold J Statsmodels: econometric and statistical modeling with python.

[40] Lindstrom MJ, Bates DM (1988). Newton—Raphson and EM algorithms for linear mixed-effects models for repeated-measures data. J Am Stat Assoc.

[41] Kuznetsova A, Brockhoff PB, Christensen RHB (2017). lmeTest package: tests in linear mixed effects models. J Stat Softw.

[42] Westfall J, Kenny DA, Judd CM (2014). Statistical power and optimal design in experiments in which samples of participants respond to samples of stimuli. J Exp Psychol Gen.

[43] Brysbaert M, Stevens M (2018). Power analysis and effect size in mixed effects models: a tutorial. J Cogn.

[44] Steghaus S, Poth CH (2022). Assessing momentary relaxation using the Relaxation State Questionnaire (RSQ). Sci Rep.

[45] Davis FD, Granić A (2024). The Technology Acceptance Model - 30 Years of TAM.

[46] Davis FD, Bagozzi RP, Warshaw PR (1989). Technology acceptance model. J Manag Sci.

[47] Fleming T, Sutcliffe K, Lucassen M, Pine R, Donkin L (2022). Serious Games and Gamification in Clinical Psychology.

[48] Fish MT, Saul AD (2019). The gamification of meditation: a randomized-controlled study of a prescribed mobile mindfulness meditation application in reducing college students’ depression. Simul Gaming.

[49] Victorson D, Kentor M, Maletich C (2015). Mindfulness meditation to promote wellness and manage chronic disease. Am J Lifestyle Med.

[50] Asselmann E, Zenker M, Rückert F, Kische H, Pieper L, Beesdo-Baum K (2023). Ecological momentary assessment and applied relaxation: results of a randomized indicated preventive trial in individuals at increased risk for mental disorders. PLoS ONE.

[51] Perciavalle V, Blandini M, Fecarotta P (2017). The role of deep breathing on stress. Neurol Sci.

[52] Ma X, Yue ZQ, Gong ZQ (2017). The effect of diaphragmatic breathing on attention, negative affect and stress in healthy adults. Front Psychol.

[53] Epe J, Stark R, Ott U (2021). Different effects of four yogic breathing techniques on mindfulness, stress, and well-being. OBM Integr Complement Med.

[54] CanRelax: effectiveness of a mindfulness and relaxation self-care app on distress of people with cancer. OSF.

