# Supplementary material for: Long-Term Usage of Breeze, a Gamified Breathing Training App, and Its Effect on Momentary Relaxation in People With Cancer: Cohort Study
Source: JMIR Serious Games. 2025 Dec 16;13:e70297. doi: 10.2196/70297 (PMC12707441; doi:10.2196/70297)

## Multimedia Appendix 1. Cohort Analysis

**Figure 1.** Co-occurrence of study participants in different weeks. Unique participants are displayed in the diagonal cells. Example read: row 1, column 1: 58 unique participants used Breeze in Week one. row 3, column 6: 12 participants from week 3 returned in week 6.


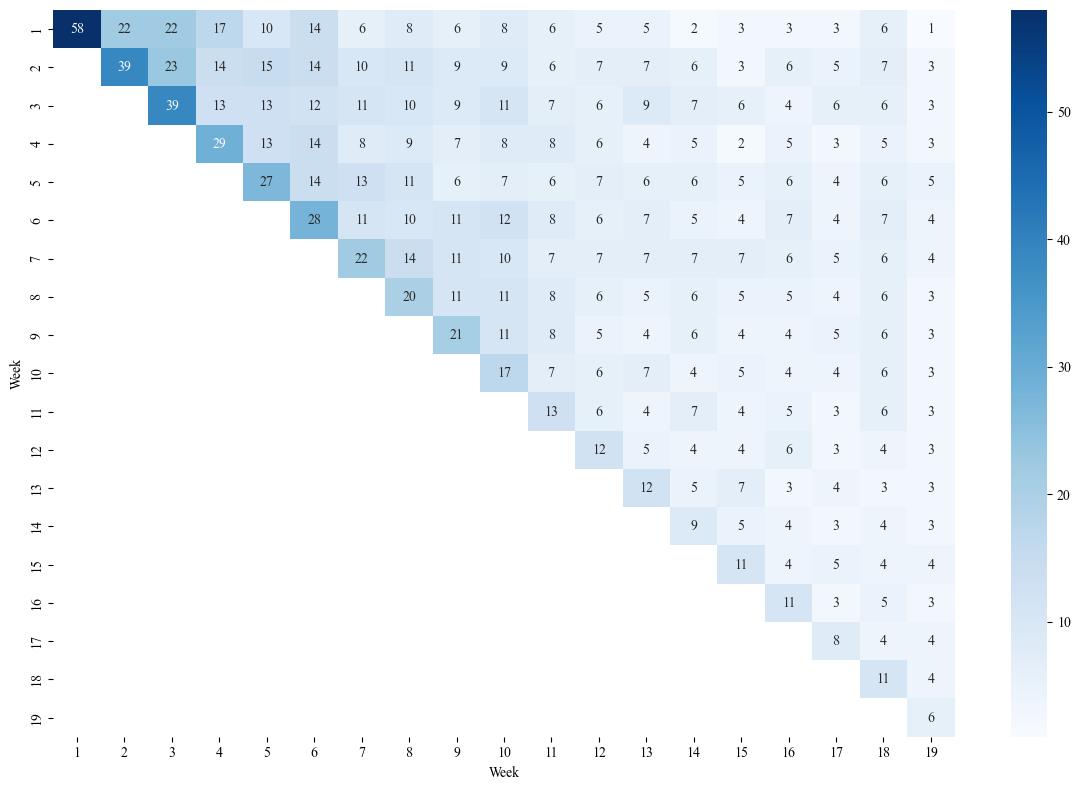


**Figure 2.** Weekly Breeze sessions per participant. Each cell represents the number of Breeze sessions performed per participant per week.


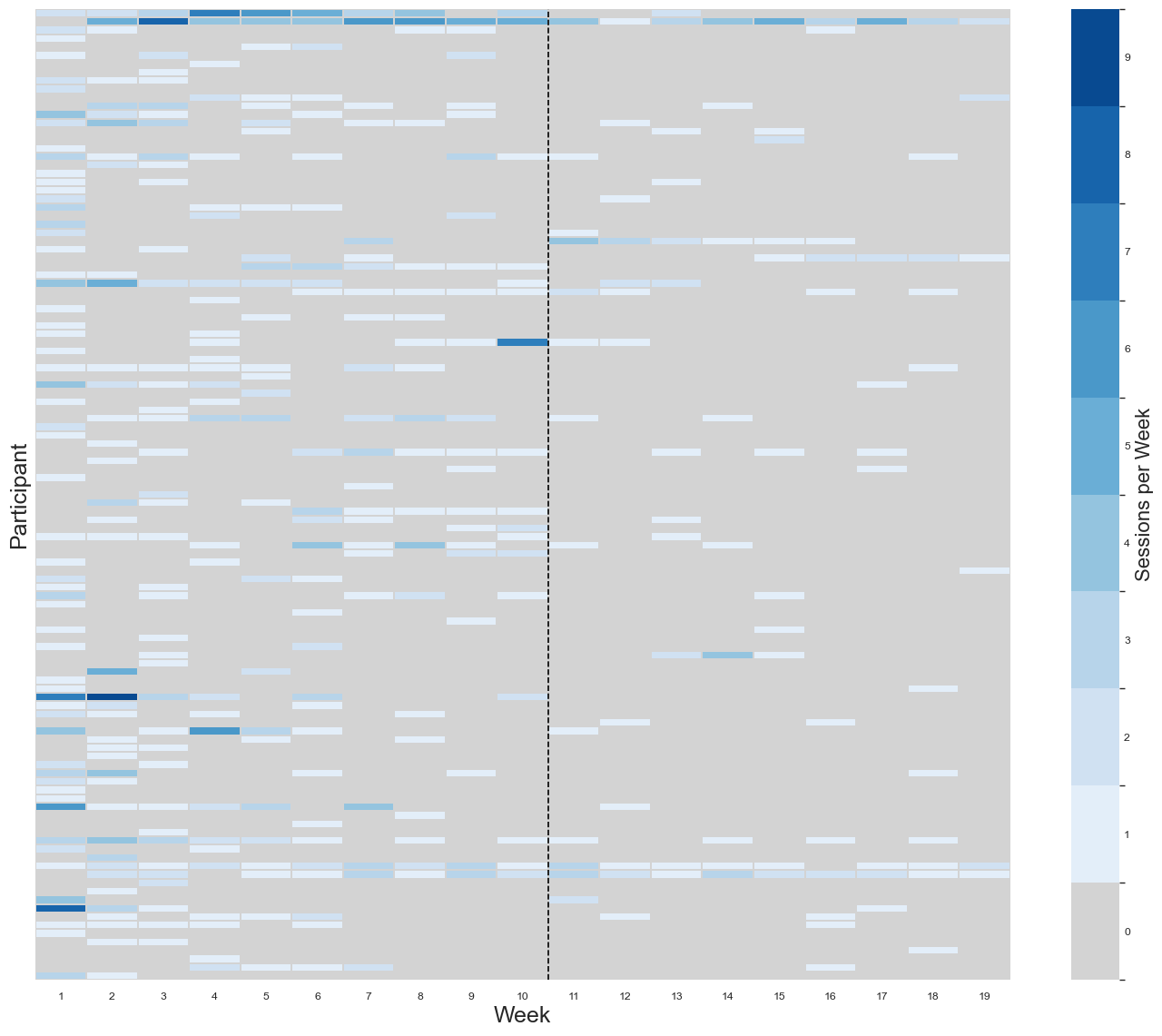

Supplement: Multimedia Appendix 1 [file games-v13-e70297-s001.docx]
