# Supplementary material for: Long-Term Usage of Breeze, a Gamified Breathing Training App, and Its Effect on Momentary Relaxation in People With Cancer: Cohort Study
Source: JMIR Serious Games. 2025 Dec 16;13:e70297. doi: 10.2196/70297 (PMC12707441; doi:10.2196/70297)
Supplement: Multimedia Appendix 2 [file games-v13-e70297-s002.docx]

## Multimedia Appendix 2: Momentary Relaxation Effect of Non-Breeze Exercises

Overview of the effect and time of non-Breeze exercises in the CanRelax 2.0 app. The intervention effect is calculated the same way as for Breeze in Table 2. N represents the number of pre-post pairs. Moreover, the default exercise duration, its mean and standard deviation are provided, too.

| **Exercise** | **Effect** | ***Effect SD*** | ***P* value** | **d** | **N** | **Default (min)** | **Mean (min)** | **SD (min)** |
| --- | --- | --- | --- | --- | --- | --- | --- | --- |
| Mindfulness meditation | -1.30 | 0.16 | < 0.001 | -0.56 | 165 | 15 | 15.0 | 0 |
| Body Scan | -1.38 | 0.23 | < 0.001 | -0.60 | 93 | 35 | 36.8 | 1.1 |
| Walking Meditation | -0.64 | 0.27 | .018 | -0.28 | 67 | 8 | 7.8 | 0.4 |
| Guided Imagery | -1.17 | 0.20 | < 0.001 | -0.50 | 140 | 15 | 15.1 | 0.1 |
| Short Meditation | -1.12 | 0.13 | < 0.001 | -0.49 | 241 | 5 | 5.0 | 0 |
| Progressive Muscle Relaxation | -1.44 | 0.24 | < 0.001 | -0.64 | 93 | 12.5 | 12.6 | 0.1 |
| Breeze (2 min) | -0.42 | 0.11 | < 0.001 | -0.19 | 249 | 2 | 2.5 | 0.68 |
